# Portuguese translation and validation of the questionnaires from the Canadian Physical Literacy Assessment-2: a pilot study

**DOI:** 10.3389/fpsyg.2023.1244566

**Published:** 2023-11-17

**Authors:** María Mendoza-Muñoz, Jorge Carlos-Vivas, Antonio Castillo-Paredes, Jose A. Parraca, Armando Raimundo, Joana Alegrete, Raquel Pastor-Cisneros, Rafael Gomez-Galan

**Affiliations:** ^1^Research Group on Physical and Health Literacy and Health-Related Quality of Life (PHYQOL), Faculty of Sport Sciences, University of Extremadura, Cáceres, Spain; ^2^Departamento de Desporto e Saúde, Escola de Saúde e Desenvolvimento Humano, Universidade de Évora, Évora, Portugal; ^3^Physical Activity for Education, Performance and Health (PAEPH) Research Group, Faculty of Sport Sciences, University of Extremadura, Cáceres, Spain; ^4^Grupo Investigación en Actividad Física y Salud Escolar, Escuela de Pedagogía en Educación Física (AFySE), Facultad de Educación, Universidad de Las Américas, Santiago, Chile; ^5^Comprehensive Health Research Centre (CHRC), University of Évora, Évora, Portugal; ^6^Promoting a Healthy Society Research Group (PheSo), Faculty of Sport Sciences, University of Extremadura, Cáceres, Spain; ^7^Research Group on Physical and Health Literacy and Health-Related Quality of Life (PHYQOL), University of Extremadura, Mérida, Spain

**Keywords:** child, cross-cultural adaptation, physical education, validity, CAPL-2

## Abstract

**Background/objective:**

Physical literacy assessment is considered a vital resource to decrease the prevalence of sedentary lifestyles and physical inactivity in children and adolescents worldwide. In Portugal, there is no physical literacy assessment tool for children under 15 years old. The main objective of this study was to carry out a translation and cultural adaptation of the Canadian Physical Literacy Assessment 2 (CAPL-2) into Portuguese, as well as to test its psychometric properties, in children between 8 and 12 years of age.

**Methods:**

The questionnaires included in the CAPL-2 were translated using the translation-back-translation method and adapted to their context. The test–retest reliability, internal consistency, and confirmatory factor analysis of the CAPL-2 Portuguese version were analyzed in a sample of 69 and 138 students, respectively, from a school in the Alentejo region (Portugal).

**Results:**

The Portuguese version of the CAPL-2 questionnaires demonstrated high internal consistency (Cronbach’s α: 0.713–0.979) and test–retest reliability ranging from moderate to nearly perfect in the motivation and confidence domain and knowledge and comprehension domain (ICC = 0.549–0.932). The results showed a good fit after adjusting for covariation paths (CMIN/DF = 1.382, *p* = 0.105, RMSEA = 0.053, CFI = 0.971, TLI = 0.955, NFI = 0.907).

**Conclusion:**

The CAPL-2 version of the questionnaires, translated and adapted to the Portuguese context, demonstrated validity and reliability, making them suitable for assessing physical literacy in children aged 8–12 years.

## 1 Introduction

The World Health Organisation (WHO) points out that childhood obesity is one of the main problems for public health and a great challenge for our society, as it has increased notably in recent years (WHO, n.d.). In Portugal, studies (Ferreira and Marques-Vidal, 2008; Frade et al., 2020) that have evaluated the prevalence of overweight and obesity have revealed that the prevalence of overweight ranges between 20 and 40% and obesity between 10 and 15% in children and adolescents (Frade et al., 2020). In contrast, the Childhood Obesity Surveillance Initiative (COSI) in Portugal observed a reverse trend in the period from 2008 to 2019 indicating the prevalence of overweight and obesity in children aged 6–8 years (Luo et al., 2019). However, the data are worrying, as the National Survey on Food, Nutrition and Physical Activity 2015–2016 (IAN-AF, 2015–2016) found that the prevalence of overweight and obesity in children was 25%, and even higher in adolescents, reaching 32.3% (Oliveira et al., 2018).

Sedentary lifestyles, metaphorically considered the disease of today’s society, have increased markedly and are the main cause of the rise in childhood overweight and obesity (Matamoros, 2019; Rodulfo, 2019). The IAN-AF, 2015–2016 (Oliveira et al., 2018) reported that 57.5% of Portuguese children and adolescents (aged 6–14 years) met the recommendations set by the WHO [60 min/day of moderate/vigorous physical activity (PA) (≥3METS/h)]. Within this group, children are more likely to meet the recommendations than adolescents.

Considering the increase in the prevalence of overweight and obesity in children and adolescents as well as the trend of non-compliance with PA recommendations by adolescents, school age is a key time to curb it. Therefore, due to the consequences of physical inactivity and the risk it poses to health in the short and long term, physical literacy (PL) could play a key role, as it is defined as the motivation, confidence, physical competence, knowledge, and understanding of value and participation in a physically active lifestyle (de Balazs et al., 2017; Cairney et al., 2019).

With regard to health, although the influence of PL on health has been recognized, the extent of its importance and implications in this area is not yet known. Research that has studied the relationship between PL and health is very scarce; however, data have been reported that it is related to aspects such as body composition (Mendoza-Muñoz et al., 2020), physical fitness, blood pressure and health-related quality of life (Caldwell et al., 2020).

This has led to a growing interest in PL monitoring (Tremblay and Lloyd, 2010), possibly because the results of the evaluations can be very useful for teachers to adapt their school plan, principals to try to obtain more resources for the improvement of PL, and public administrations to raise awareness of the importance of PL among policy makers and, in turn, to allocate resources for its proper development (Tremblay and Lloyd, 2010). The evaluation of PL can also allow the detection of specific deficiencies in the different domains that compose it, to orientate the appropriate interventions for its full development, thus allowing a follow-up or monitoring of the same, both to know the effects of specific programs and to explore its evolution over time. In this context, a review has shown numerous investigations that have attempted to monitor PL in different ways in each of its domains (Shearer et al., 2021). However, worldwide for children, we found only three specific and validated PL tools: the Canadian Physical Literacy Assessment (Longmuir et al., 2018), Passport for Life (PPL) (Lodewyk and Mandigo, 2017), and PlayFun (Cairney et al., 2018; Stearns et al., 2019), and a study that is under development, such as The Portuguese Physical Literacy Assessment (PPLA) (Mota et al., 2021).

The Canadian Assessment of Physical Literacy (CAPL) (Longmuir et al., 2018) was the first tool used for the measurement of physical literacy data. This tool includes the domains of fundamental motor skills, physical activity behavior, physical fitness, and physical activity knowledge and understanding. Currently, this tool is one of the most widely used worldwide to assess PL in children and adolescents, and has recently been used in studies in Denmark (Elsborg et al., 2021), China (Li et al., 2020), Greece (Dania et al., 2020), Iran (Valadi and Cairney, 2022), and Spain (Mendoza-Muñoz et al., 2021; Pastor-Cisneros et al., 2022). The studies that have validated this tool in different countries have shown through confirmatory factor analysis that its model fit acceptably with the original model (Dania et al., 2020; Li et al., 2020; Elsborg et al., 2021).

In Portuguese, there is only one tool for the evaluation of PL “PPLA”(Mota et al., 2021; Rodrigues, 2022), but this tool is intended for children between 15 and 18 years of age.

Considering that, currently, the CAPL is the most complete and widely used generic tool to assess physical literacy worldwide and that it is intended for children from 8 to 12 years of age, it has been considered the assessment battery chosen to be applied in Portuguese children.

Therefore, because of the growing interest in the study of PL (Lundvall, 2015; Edwards et al., 2017) and its monitoring, as well as the lack of assessment instruments to evaluate it in schoolchildren, this study aimed to translate and culturally adapt the Canadian Physical Literacy Assessment 2 into Portuguese and to test its psychometric properties in children between 8 and 12 years of age.

## 2 Methods

### 2.1 Study design

A pilot study was conducted on a cross-cultural adaptation of the questionnaires of the Canadian Physical Literacy Assessment 2 and cross-sectional validation study was carried out.

### 2.2 Ethics approval

The Ethics Committee of the University of Évora was approved this proyect (approval number: 22047), adhering to the last revision of the Declaration of Helsinki and modified by the 64th General Assembly of the World Medical Association (Fortaleza, Brazil, 2013) and in accordance with the provisions outlined in Law 14/2007 on Biomedical Research.

### 2.3 Measures

The questionnaires that underwent the process of translation and cultural adaptation belonged to the Canadian Physical Literacy Assessment-2 battery (Longmuir et al., 2018). This assessment has been used in many countries (Dania et al., 2020; Elsborg et al., 2021; Mendoza-Muñoz et al., 2021; Valadi and Cairney, 2022), as it is one of those that best fits the concept of children’s PL, as it is composed of four domains: (1) daily physical activity behavior (DB); (2) physical competence (PC); (3) motivation and confidence (MC); and (4) knowledge and understanding (KU). Each domain was evaluate throught the utilization of various questionnaires and tests, which are outlined in Table 1.

**Table 1:** Canadian Assessment of Physical Literacy (CAPL-2) questionnaires and tests (Longmuir et al., 2018).

Domain	**Questionnaires forming part of the validation process of the study**	**Other tests that are part of the domain**
Daily physical activity behavior (DB)	Self-reported question on minutes of physical activity per week performed	Xiaomi mi Band 3 (Xiaomi Corporation, Pekín, China)
Physical competence (PC)		Canadian Agility and Movement Skill Assessment (CAMSA) (Longmuir et al., 2017) Plank isometric hold (Boyer et al., 2013) Progressive Aerobic Cardiovascular Endurance Run (PACER) (Scott et al., 2013)
Motivation and confidence (MC)	Motivation and confidence questionnaires of the Canadian Assessment of Physical Literacy Development (CAPL-2) (Longmuir et al., 2018)	
Knowledge and understanding (KU)	Questionnaires on the knowledge and understanding of the Canadian Assessment of Physical Literacy Development (CAPL-2) (Longmuir et al., 2018)	

### 2.4 Procedures

The tests in bold are those that form a part of the questionnaire to be translated, culturally adapted, and validated in Portuguese.

Specifically, the questionnaires belonging to each domain were scored as follows:

DB: The questionnaire associated with this specific domain contained an inquiry designed to ascertain the frequency of participants engaging in at least 60 min of physical activity per day. Responses to this question were rated on a scale of 1 to 5, with 1 representing the lowest level of daily activity and 5 the highest. The daily activity domain will, therefore, consist of this question, together with the total steps taken during a week (25 points), making a total of 30 points out of the total PL (100 points).

MC: This domain consists of a questionnaire designed to evaluate participants’ self-assurance in engaging in PA and their motivation to partake in such activities (Longmuir et al., 2018). The overall score (30 points) is calculated by combining scores from four dimensions (intrinsic motivation, competition, predilection, and appropriateness). Each dimension is scored on a scale ranging from 1 to 7.5 points, and the four domains can be summed up to a total of 30 points. A score of 1 represents the lowest possible rating, while a score of 30 indicates the highest attainable rating.

KU (Longmuir et al., 2018): This instrument assesses knowledge concerning PA. More specifically, the questionnaire consisted of four multiple-choice questions with four answer options, with a value of 1 point for each correct answer and a text-type question where you must fill in the blanks to complete a story. Each correctly filled gap was assigned one point up to a total of 6. That is, the sum of the multiple-choice questions plus the sum of the text questions will make a possible score of up to 10 points, with 1 being the lowest possible knowledge of PA and 10 being the highest.

#### 2.4.1 Phase 1: translation and cultural adaptation of the Portuguese version of the questionnaires belonging to Canadian Physical Literacy Assessment-2

**Figure 1:**
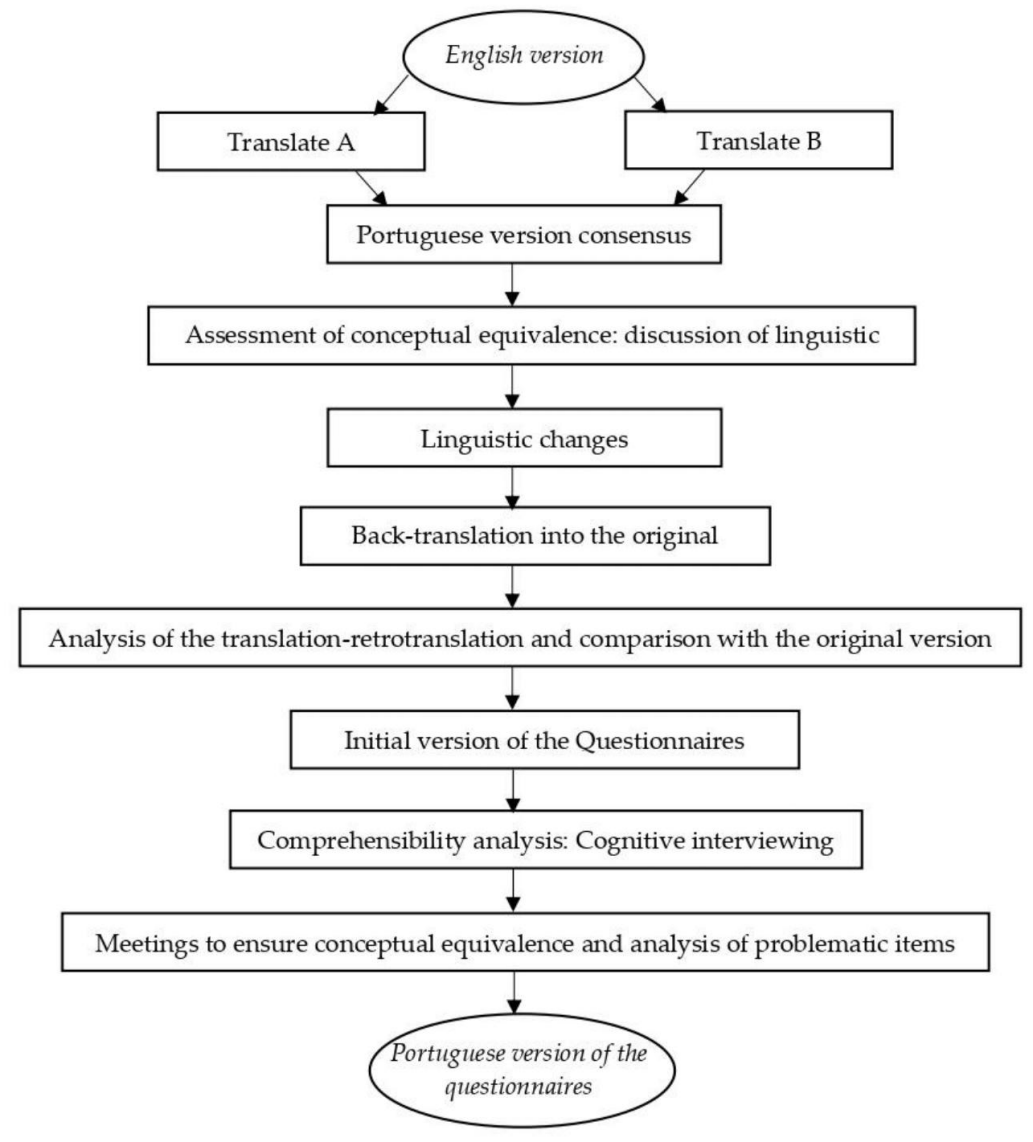
Procedure for translation and culture adaptation for the Portuguese version of the questionnaires belonging to the Canadian physical Literacy Assessment-2.

Before beginning the study, permission was sought from one of the original researchers to begin the process of translation and cultural adaptation. Phase 1 began with the translation of both questionnaires by two Portuguese translators who are expert researchers in Sports Sciences and Physical Education, with a command of the original language (English). These translators were asked to rate the level of difficulty they encountered while translating each question on a scale of 0 to 10, with 0 indicating no difficulty and 10 representing significant difficulty. Once each translator had produced an individual version of the questionnaire, a consensus meeting was conducted to reach a unified translation and cultural adaptation of the questionnaires. During this meeting, linguistic differences were discussed, leading to the creation of the initial version of the questionnaire. Subsequently, a process of back-translation was carried out. This involved translating the previously translated Portuguese version of the questionnaire back into the original language (English). This translation was carried out by a translator who was a native English speaker fluent in Portuguese and expert in Sports Sciences and Physical Education. Once the back-translation was obtained, a comparison of this version with the original (English) was carried out, resulting in a correctly translated questionnaire (Figure 1).

The Portuguese version was evaluated in terms of comprehension in a sample of 18 students aged between 8 and 12 years from different sports schools in Évora (Portugal). The participants underwent face-to-face interviews (Conrad et al., 1999). This interview aimed to detect possible errors and/or misunderstandings in the questionnaire, as well as suggestions for improvement.

The interviews were conducted by the same person in Portuguese, and the following items were evaluated. First, the comprehension of the items was evaluated using a three-point ordinal scale: (1) clear and understandable, (2) difficult to understand, and (3) incomprehensible. Subsequently, the items were assessed using a numerical scale ranging from 0 to 10, where 0 denoted a high level of ease in comprehension and 10 indicated significant difficulty in understanding. Finally, the respondents were asked to express the perceived meaning of the questionnaire items in their own words.

The questionnaire was generally evaluated by the participants as clear and understandable, although some minor cultural adaptations were made and agreed upon. The final version of the questionnaire was obtained from interviews.

#### 2.4.2 Phase 2: psychometric properties of the questionnaire

For this phase, test–retest reliability and internal consistency and Confirmatory Factorial Analyses (CFA) were carried out to assess the psychometric properties of the questionnaire, following other research (Wang et al., 2016; Feng et al., 2023).

##### 2.4.2.1 Test–retest reliability and internal consistency

In order to evaluate the test–retest reliability and internal consistency of the questionnaires, a total of 69 participants were recruited from various schools in Évora, Portugal, through convenience sampling. After obtaining the final versions of the questionnaires, permission was sought from the school principals to conduct the study. Subsequently, parental consent was obtained for their children’s participation in the research. Then, the children signed an informed consent form and agreed to participate in this study. Once permission had been obtained from all parties and the children had agreed to participate, each participant completed the final questionnaires in their Portuguese version on paper, with an interval between the first and second measurements of 21 days. In addition, sociodemographic data such as age and sex were collected.

##### 2.4.2.2 Confirmatory factorial analyses

Subsequently, a total of 138 children completed the final version of the questionnaire, which was subjected to CFA.

### 2.5 Statistical analyses

The IBM SPSS Statistics 25 software was used for statistical analyses. All the collected information was entered into a dedicated database for this research, with a stringent focus on maintaining the anonymity of personal information. Participants with missing data were excluded prior to start any analysis. Data are showed as mean and standard deviation and median and interquartile range. The Kolmogorov–Smirnov and Levene’s test were, respectively, applied to check the normality and homogeneity of data.

The internal reliability of the total scale and the internal consistency of every item and domain were determined using the Cronbach’s coefficient. Cronbach’s alpha was interpreted as follows Glen (2022): <0.5, unacceptable; ≥0.5 to <0.6, poor; ≥0.6 to <0.7, questionable; ≥0.7 to <0.8, acceptable; ≥0.8 to <0.9, good; and >0.9, excellent. McDonald’s omega coefficient was also calculated as composite reliability outcome (Hayes and Coutts, 2020). Relative reliability was determined using the intraclass correlation coefficient (ICC 3,1) (Shrout and Fleiss, 1979), and the standard error of measurement (SEM) and minimum true difference (MDR) (Weir, 2005) were used to define absolute reliability. A test–retest of the two assessments, 21 days apart, was conducted. The alpha level was set at *p* ≤ 0.05. The ICC was classified according to Portney and Watkins (2000): ICC <0.50, low reliability, 0.50 to 0.75 moderate, 0.75 to 0.90, good; and > 0.9, excellent.

The confirmatory factor analysis (CFA) was conducted through the software package AMOS v.18.0.0 (IBM Corporation, Wexford, PA, USA). The different domains and items obtained from the previous EFA were included as elements. To evaluate the adequacy of the model’s fit, several indices were utilized, including: (1) the chi-square probability setting as appropriate non-significant values (*p* > 0.05) (Green et al., 1997), (2) the root mean square error of approximation (RMSEA;) (Xia and Yang, 2019), (3) the comparative fit index (CFI), (4) the Tuker-Lewis index (TLI), (5) the normed fit index (NFI), and (6) the chi-square per degree of freedom ratio (CMIN/DF) (Wells, 2021). Regarding RMSEA, values of 0.01, 0.05 and 0.08 indicate excellent, good and mediocre fit respectively, some go up to 0.10 for mediocre. Likewise, for CFI, TLI and NFI fit indexes, values over 0.90 reflect a good fit (Schumacker and Lomax, 2004). Finally, if the CMIN/DF value is ≤3 it indicates an acceptable fit (Kline, 2016).

Additionally, an invariance analysis was performed to check the stability of the psychometric properties across the different groups, based on sex. Two criteria were contrasted (Cheung and Rensvold, 2002; Byrne, 2013): (1) the model should be adjusted for each group and (2) four types of invariance should be examined: configural (the same item should be associated with the same factor in each group), metric (compares regression slopes or score changes), scalar (the scores of the different groups have the same unit of measurement and the same origin) and residual (group differences in the items are due only to differences in the factors). The values recommended in the literature are (Chen, 2007): changes of 0.01 for CFI (ΔCFI) and changes of 0.015 for RMSEA (ΔRMSEA).

## 3 Results

### 3.1 Phase 1: translation and cultural adaptation of the Portuguese version of the questionnaires belonging to Canadian Physical Literacy Assessment-2

In the initial version (Version 1) of the questionnaire, prior to the back-translation process, certain words, concepts, and terms were modified through consensus, as outlined in Table 2.

**Table 2:** Modifications were incorporated during the transition from the initial translated version to the final version of the questionnaire.

**Original version (English)**	**Agreed version of the translations**	**Adaptations of the translated version (first consensus version)**	**Version after cognitive interviews**
Beat faster	bata mais rápido	bata mais depressa	
Try	Tenta	Experimenta	
Circled	Uma bola	Um circulo	
Boys and girls	Meninos e meninas	Rapazes e rapazigas	
Not true for me	Não é verdade para mim		Discordo muito
Not really true for me	Não é realmente verdade para mim		Discordo
Sometimes true for me	Por vezes é verdade para mim		Concordo
Often true for me	Muitas vezes é verdade para mim		Concordo Bastante
Very true for me	É muito verdadeiro para mim		Concordo totalmente
kinds of fitness	Condições física	Aptidão física	
Good	Bom	Boa	
Breathe hard	Respirar mais ráprido	respirar com dificuldade	
Really true for me	realmente verdadeiro		Concordo totalmente
Sort of true for me	meio verdadeiro		Concordo mais ou menos
Mau	Mau	Mau	Ruim
Sally	Sally	Maria	

Subsequently, this version was subjected to back-translation and a comparison was made between this version and the original (English) version. After extensive deliberation, the researchers reached a consensus and found no significant disparities between the two versions.

To conclude this phase, cognitive interviews were conducted with a total of 18 participants (10 girls and 8 boys), with a mean age of 10.10 (±1.41) years. The participants provided positive feedback regarding their comprehension of the questionnaires. No difficulties in understanding the instructions and items were reported by the participants, as they unanimously rated the questionnaires as clear and easily comprehensible. However, minor adjustments were made based on their suggestions, specifically addressing two statements and one word, as shown in Table 2.

Following these modifications, the final version of the questionnaire was attained.

### 3.2 Phase 2: psychometric properties of the questionnaire

#### 3.2.1 Test–retest reliability and internal consistency

A total of 69 children (10.48 ± 0.61 years old) participated during the phase, of whom 39 (56.5%) were boys and 30 girls (43.5%).

Tables 3–5 show the internal consistency, reproducibility, and systematic differences between the self-reported PA, MC, and knowledge and understanding questions of the CAPL-2 battery, respectively. Overall, great internal consistency was observed for all questions, domains, and total scores of the questionnaires (Cronbach’s α ranged from 0.713 to 0.979). Significant correlations were found for all items in the test and retest with the total score of their domain (*r* > 0.332).

**Table 3:** Reliability, test–retest and systematic differences of auto-reported physical activity question from CAPL-2 battery assessment.

	Test (*n* = 69)			Retest (*n* = 69)			Reliability test						
	M	SD	Item-total correlation	*M*	SD	Item-total correlation	Cronbach’s α	ICC (95% CI)	Value of *p*†	SEM	%SEM	MDC	MDC %
DB	4.29	1.83	N/A	4.35	1.89	N/A	0.979	0.958	0.371	0.38	8.8	1.06	24.4

DB, Daily Physical Activity Behavior; M, Mean; SD, standard deviation; 95% CI, confidence interval of 95%; ICC, intraclass correlation coefficient; SEM, standard error of measurement; %SEM, standard error of measurement as a percentage; MDC, minimum detectable change; N/A, not applicable; ^†^Friedman test value of *p*s. Item-total correlation refers to the magnitude of association between each item with its domain.

**Table 4:** Reliability, test–retest, and systematic differences in motivation and confidence questions from the CAPL-2 battery assessment.

MC	Test (*n* = 69)			Retest (*n* = 69)				Reliability test					
	*M*	SD	Item-total correlation	*M*	SD	Item-Total Correlation	Cronbach’s α	ICC (95% CI)	Value of *p*†	SEM	%SEM	MDC	MDR%
Item 1	2.27	0.52	0.518**	2.28	0.51	0.436**	0.914	0.844	0.770	0.20	8.9	0.56	24.8
Item 2	2.27	0.55	0.396**	2.24	0.55	0.337**	0.900	0.820	0.444	0.23	10.3	0.65	28.6
Item 3	2.25	0.60	0.497**	2.17	0.60	0.364**	0.940	0.881	0.027	0.21	9.4	0.57	25.9
Predilection	6.79	1.21	0.772**	6.69	1.25	0.734**	0.934	0.920	0.890	0.35	5.2	0.96	14.3
Item 1	2.19	0.56	0.474**	2.25	0.49	0.566**	0.874	0.775	0.207	0.25	11.2	0.69	31.0
Item 2	2.07	0.63	0.692**	2.03	0.63	0.686**	0.933	0.875	0.287	0.22	10.9	0.62	30.2
Item 3	2.07	0.67	0.523**	2.07	0.62	0.677**	0.943	0.894	0.903	0.21	10.1	0.58	28.1
Adequacy	6.56	1.02	0.773**	6.35	1.28	0.875**	0.713	0.549	0.118	0.78	12.0	2.15	33.3
Item 1	2.19	0.41	0.592**	2.21	0.42	0.525**	0.903	0.823	0.467	0.18	8.0	0.49	22.2
Item 2	2.21	0.38	0.601**	2.22	0.37	0.529**	0.831	0.713	0.670	0.20	9.0	0.55	25.0
Item 3	2.16	0.47	0.602**	2.17	0.48	0.551**	0.936	0.881	0.796	0.16	7.6	0.46	21.1
Intrinsic Motivation	6.56	1.02	0.773**	6.60	1.06	0.673**	0.945	0.896	0.446	0.34	5.1	0.93	14.1
Item 1	1.78	0.51	0.676**	1.90	0.49	0.654**	0.919	0.836	0.007	0.20	11.0	0.56	30.5
Item 2	1.77	0.61	0.683**	1.80	0.57	0.684**	0.931	0.871	0.317	0.21	11.9	0.59	33.1
Item 3	2.04	0.54	0.643**	2.05	0.47	0.672**	0.932	0.874	0.637	0.18	8.8	0.50	24.4
Physical Activity Competence	5.60	1.45	0.816**	5.75	1.33	0.801**	0.948	0.898	0.057	0.45	7.8	1.23	21.7
Total MC domain score	25.29	3.93	N/A	25.74	4.68	N/A	0.908	0.932	0.141	1.12	4.4	3.11	12.2

MC, motivation and confidence; M, mean; SD, standard deviation; 95% CI, confidence interval of 95%; ICC, intraclass correlation coefficient; SEM, standard error of measurement; %SEM, standard error of measurement as a percentage; MDC, minimum detectable change; N/A, not applicable; ^†^Friedman test value of *p*s; **p < 0.01 for item-total correlation. Item-total correlation refers to the magnitude of the association between each item and its domain.

**Table 5:** Reliability, test–retest, and systematic differences in knowledge and understanding questions from the CAPL-2 battery assessment.

KU	Test (*n* = 69)			Retest (*n* = 69)				Reliability test					
	*M*	SD	Item-total correlation	*M*	SD	Item-total correlation	Cronbach’s α	ICC (95% CI)	*P*-value^†^	SEM	%SEM	MDC	MDC%
Physical activity (PA) guidelines	0.51	0.50	0.332**	0.64	0.48	0.349**	0.737	0.567	0.020	0.33	56.8	0.90	157.4
Cardiorespiratory fitness definition	0.72	0.45	0.546**	0.78	0.41	0.408**	0.820	0.692	0.157	0.24	31.9	0.67	88.3
Muscular endurance definition	0.75	0.43	0.409**	0.83	0.38	0.353**	0.765	0.614	0.096	0.25	32.1	0.70	88.9
Improve sport skills	0.43	0.50	0.398**	0.45	0.50	0.390**	0.848	0.738	0.739	0.26	57.9	0.71	160.6
PA comprehension	4.77	1.15	0.798**	4.81	1.09	0.810**	0.894	0.809	0.602	0.49	10.2	1.36	28.3
Total KU domain score	7.19	1.72	N/A	7.51	1.55	N/A	0.885	0.781	0.015	0.76	10.4	2.12	28.8

KU, knowledge and understanding; M, mean; SD, standard deviation; 95% CI, confidence interval of 95%; ICC, intraclass correlation coefficient; SEM, standard error of measurement; %SEM, standard error of measurement as a percentage; MDC, minimum detectable change; N/A, not applicable; ^†^Friedman test *p*-values; **p < 0.01 for item-total correlation. Item-total correlation refers to the magnitude of the association between each item and its domain.

Reproducibility results revealed near-perfect test–retest reliability for the self-reported physical activity behavior question (ICC = 0.958) and moderate to near-perfect test–retest reliability for each domain and total score of the motivation and confidence domain (ICC = 0.549–0.932); as well as for the KU domain and items (ICC = 0.567–0.809). The SEM and SEM% ranged from 0.16 to 1.06 and from 4.2 to 57.9, respectively. The MDC varied from 0.46 to 2.93.

Finally, the test–retest comparison revealed no significant differences for all items and domains (*p* > 0.05), except for the third item of the predilection subdomain (*p* = 0.027) and the Item 1 (*p* = 0.020), and the total score of the KU domain (*p* = 0.015).

#### 3.2.2 Confirmatory factorial analyses

**Figure 2:**
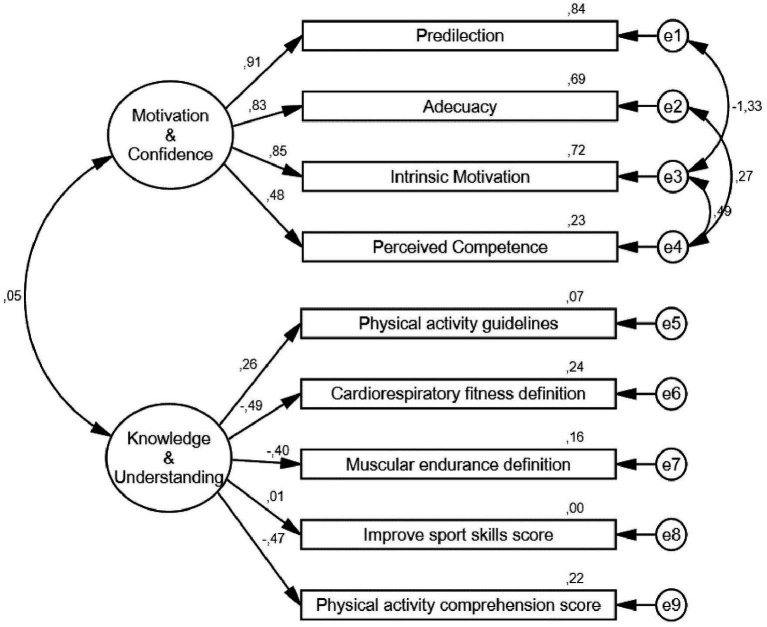
CFA resulting model for Motivation & Confidence and knowledge and Understanding domains of Portuguese version.

A confirmatory factor analysis was conducted with a total of 138 participants aged 10.46 (±0.66) years, of whom 52.2% were boys and 47.8% were girls. Figure 2 presents the resulting model from the CFA.

Table 6 illustrates the goodness-of-fit indices after CFA (Kassim et al., 2013). The chi-square probability was not significant (*p* = 0.105), and the RMSEA was acceptable (0.050–0.080) (Lt and Bentler, 1999). Likewise, the rest of the goodness-of-fit indices revealed a good fit between the data and model (Schumacker and Lomax, 2004). The CMIN/DF index reveals good values, since it must be below 3 for a correct model fit, and the CFI, TLI, and NFI indices are over 0.9 which indicates a close-to-perfect fit to the model. Moreover, good composite reliability was also reported for the intruments (McDonald’s ω = 0.735).

**Table 6:** Motivation and Confidence and Knowledge and Understanding questionnaires – Portuguese version goodness-of-fit indices.

**Indices**	**Value**
CMIN/DF	1.382
P (CMIN)	0.105
RMSEA	0.053
CFI	0.971
TLI	0.955
NFI	0.907

CMIN/DF, minimum discrepancy per degree of freedom; P (CMIN), chi-squared probability; RMSEA, root mean square error of approximation; CFI, comparative fit index; TLI, Tuker-Lewis index; NFI, normed fit index.

In addition, a multi-group CFA was conducted to test for measurement invariance (Table 7). Initially, we tested the configuration invariance model, baseline or free (M1), which proposed that the instrument would have a bifactorial structure in all sex groups and allowed factor loadings, intercepts and error variances to be freely estimated. The indices obtained (CFI = 0.985; RMSEA = 0.019; CMIN/GL = 1.096) indicated that the fit of the model to the data was adequate. The metric invariance model (M2) was then tested, in which the factor loadings were constrained to be equal regardless of sex. The indices showed that the model fitted well and when compared to M1, the ΔCFI <0.01, the ΔRMSEA<0.015 and ΔCMIN was non-significant (*p* > 0.05). The scalar invariance model (M3), in which the intercepts and factor loadings were restricted to be equal between sexes, showed a good fit. When compared to M2, there were no significant changes in CFI, RMSEA or CMIN. Finally, the strict invariance model (M4), in which factor loadings, intercepts and error variances were restricted, also fitted well. In comparison with M3, the ΔCMIN was non-significant (*p* > 0.05), and the ΔRMSEA <0.015, although the ΔCFI was >0.01, contrary to expectations.

**Table 7:** Multi-group invariance analysis outcomes.

**Model**	**CMIN (DF)**	**CMIN/DF**	**CFI**	**RMSEA** **(IC 90%)**	**Comparison**	**ΔCMIN**	**ΔCFI**	**ΔRMSEA**
M1. Invariance model	109.590 (100)	1.096	0.985	0.019 (0.000–0.038)				
M2. Metric or weak invariance model (λ restricted)	116.017 (107)	1.084	0.986	0.018 (0.000–0.037)	M2 vs M1	6.427 (7), *p* = 0.491	0.001	−0.001
M3. Scalar or strong invariance model (λ y τ restricted)	132.626 (116)	1.143	0.973	0.023 (0.000–0.039)	M3 vs M2	16.609 (9), *p* = 0.055	−0.013	0.005
M4. Strict invariance model (λ, τ y θ restricted)	136.982 (131)	1.046	0.990	0.013 (0.000–0.033)	M4 vs M3	4.356 (15), *p* = 0.996	0.017	−0.010

CMIN, chi-square; DF, degrees of freedom; CMIN/DF, minimum discrepancy per degree of freedom; P, chi-squared probability; RMSEA, root mean square error of approximation; CFI, comparative fit index.

## 4 Discussion

This study aimed to translate and culturally adapt the questionnaires collected in the CAPL-2 into Portuguese, as well as to evaluate their psychometric properties for application in children aged 8 to 12 years. In phase 1, certain words, concepts and terms were modified by consensus. Subsequently, this version was back-translated and no significant disparities were found. Minor adjustments were made based on suggestions from the children participating in the cognitive interviews and finally, the final version of the questionnaire was obtained. Regarding phase 2, high internal consistency was observed for all questions, domains and total scores of the questionnaires as well as almost perfect test–retest reliability for the self-reported physical activity behavior question and moderate to almost perfect test–retest reliability for each domain and total score of the motivation and confidence domain, and for the KU domain and items. The test–retest comparison revealed no significant differences for all items and domains except for the third item of the predilection subdomain and item 1, and the total score of the KU domain. The CMIN/DF index reveals good values, indicating an almost perfect fit of the model. The instrument proved to be reliable and valid and its use as a reference for future PL assessments using this instrument is confirmed. It also provides the starting point for the full validation of the CAPL-2.

### 4.1 Main findings

The main findings of this study showed that the Portuguese version of the CAPL-2 questionnaires obtained through the linguistic validation process of direct and reverse translation following the guidelines of the World Health Organization (WHO, 2022), as well as the methodology followed by other countries that translated and adapted this tool [Denmark (Elsborg et al., 2021), China (Dania et al., 2020; Li et al., 2020), Greece (Dania et al., 2020), Iran (Valadi and Cairney, 2022) or Spain (Pastor-Cisneros et al., 2022)], was valid and reliable for its application in children aged 8 to 12 years. Specifically, the MC domain consisted of four subdomains of questions (appropriateness, preference, intrinsic motivation, and PA competence), each with three questions in total. The results showed high internal consistency (Cronbach’s α from 0.713 to 0.979) for each subdomain and total domain score, in line with the results presented in previous studies where, in the case of the Spanish population, high internal consistency was reported (Cronbach’s α from 0.730 to 0.987), as well as the Danish population (Cronbach’s α from 0.74 to 0.90) (Elsborg et al., 2021), and the Chinese population (Cronbach’s *α* = 0.082) (Dania et al., 2020; Li et al., 2020). Regarding test–retest reliability, few studies have assessed this parameter; however, the results of Pastor-Cisneros et al. (2022) (ICC = 0.720–0.981) and by Valadi and Cairney (2022) (ICC 0.90) for this domain, are similar to pre-studied in 0.881–0.932, except for 0.881–0.932, except the subdomain of adequacy which showed moderate reliability (ICC = 0.549).

Regarding the KU domain, the results showed good reliability (Cronbach’s α from 0.737 to 0.894) and moderate or acceptable internal consistency (ICC = 0.567–0.809); however, differences were detected between test and retest for the first item on daily physical activity (*p* = 0.015) as well as for the total domain (*p* = 0.020), which coincides with the study by Pastor-Cisneros, Carlos-Vivas (Pastor-Cisneros et al., 2022), where the KU domain was also significantly higher in the retest than in the test. In this sense, Dania et al. (2020) highlighted that the differences in item reliability were understandable, as children were curious about the correct answer and would look for it immediately before the next assessment day, and also that four of the five items were nominal variables (“correct”/"incorrect”) which may have statistical limitations when grouping items within this domain (Taber, 2018; Dania et al., 2020). Moreover, along these lines, the reliability for this domain in the Chinese population (*α* = 0.52) was low, as reported by Longmuir et al. (2018) for some of their items.

Regarding the validity of the presented model, CFA was only applied to the KU and MC domains because the DB domain had only one item. The model included both items from the original version (Longmuir et al., 2018) within the MC and KU domains, demonstrating that the model-generated fit statistics were satisfactory and within an acceptable range (CMIN/DF = 1.382, *p* = 0.105, RMSEA = 0.053, CFI = 0.971, TLI = 0.955, NFI = 0.907). These results were similar to previous studies that provided data on specific domain models of the CAPL-2, such as the case of Dania et al. (2020), who reported χ^2^ = 2.88, *p* = 0.718; RMSEA = 0.00; CFI = 1.000; TLI = 1.041 for the knowledge and comprehension domain and χ^2^ = 96.482, *p* < 0.01; RMSEA = 0.04; CFI = 0.844; TLI = 0.785 for the motivation and confidence domain. Similar findings were also reported in studies that included all four domains of CAPL-2 (Dania et al., 2020; Elsborg et al., 2021; Valadi and Cairney, 2022).

Overall, the results supported the good fit of the recalculants to the proposed dimensionality of the instrument tested and indicated that when the elements of the factor structure are invariant across gender, the fit indices were satisfactory, except for one of the parameters of the strict invariance models; in this case, partial invariance would be assumed (Dimitrov, 2010), although it has been acknowledged that strict invariance tests are overly restrictive (Bentler, 1995). Therefore, scores could be predominantly comparable across groups and the change in one unit would be equivalent across groups.

### 4.2 Practical applications

This study has multiple practical applications that are of great interest, as the assessment of PL in children and adolescents is essential to measure their progress and understand their level of skills and knowledge related to PA and physical education in general. Therefore, validation of this tool could be useful for professionals involved in PA, as well as future studies in Portuguese children, such as the proposed by Mendoza-Muñoz et al. (2022). Likewise, it would allow them to identify areas for improvement, establish realistic and individualized goals and objectives, monitor progress over time, and promote inclusion by helping teachers and coaches adapt programs and physical activities to ensure that children and adolescents with different abilities and levels of PL can participate and enjoy PA. Furthermore, considering that this tool is already being used in numerous countries (Dania et al., 2020; Elsborg et al., 2021; Pastor-Cisneros et al., 2022; Valadi and Cairney, 2022), the data obtained from this study can be compared with data from those countries, aiming to explore and analyze the most successful educational policies and guidelines, specifically those in which children and adolescents have the highest PL values, to guide strategies in our region in the same direction.

Moreover, it can provide useful information to parents about their children’s physical abilities and PA, which can help them promote and support an active and healthy lifestyle at home. Ultimately, this can help foster an active and healthy lifestyle and ensure that children and adolescents have the necessary skills and knowledge to maintain good physical and mental health throughout their lives. Additionally, it allows professionals to identify the skills, habits, knowledge, or motivations that children and adolescents need to improve, which can help develop specific and tailored physical education programs and plans according to their needs.

### 4.3 Limitations

A limitation of the study, as pointed out by Pastor-Cisneros et al. (2022), is that the content used in the KU domain comes from a questionnaire adapted to the Canadian context for children and adolescents. In this sense, there are numerous studies that show the social and cultural differences, especially at this age, between the American and European populations (Moselli et al., 2006; Torsheim et al., 2006; DunnGalvin et al., 2017) in terms of social values, habits, behaviors, education...etc. Therefore, there may be a discrepancy between the knowledge of Canadian and Portuguese children of these ages, as they come from totally different socio-cultural contexts, resulting in a possible lack of adaptation to the population being assessed. Hence, the direction of possible future research lies in adapting the content to the curriculum used in Portugal, thus addressing one of the most important limitations of the present study. Another limitation of the study, which is also considered the main finding, is that only the questionnaire portion of the CAPL-2 battery has been translated and adapted, as the rest of the tools used to obtain the score for each domain in this assessment battery are not questionnaires but other types of validated tests, which require another validation and adaptation process different from the one used in this study. Thus, this study represents a starting point, providing a reliable instrument for future complete validation of the battery, as other studies have already been conducted in populations such as Danish (Elsborg et al., 2021), Greek (Dania et al., 2020), Iranian (Valadi and Cairney, 2022), and Chinese (Dania et al., 2020; Li et al., 2020). In addition, convenience sampling was also used, which makes it difficult to generalize the results.

This is the first study to translate and validate the CAPL-2 questionnaires in Portuguese schoolchildren aged 8–12 years. The instrument obtained proved to be a reliable and valid tool and can be used as a reference for future assessments of PL using this instrument, as well as serving as a starting point for the complete validation of CAPL-2.

## Data availability statement

The raw data supporting the conclusions of this article will be made available by the authors, without undue reservation.

## Ethics statement

Written informed consent was obtained from the individual(s), and minor(s)’ legal guardian/next of kin, for the publication of any potentially identifiable images or data included in this article.

## Author contributions

MM-M, AR, and AC-P: conception and design of study. JP, JA, and RP-C: acquisition of data. MM-M, JC-V, and RG-G: analysis and interpretation of data. MM-M and RP-C: drafting the manuscript. JC-V and RG-G: revising the manuscript critically for important intellectual content. All authors contributed to the article and approved the submitted version.
