# Supplementary material for: Portuguese translation and validation of the questionnaires from the Canadian Physical Literacy Assessment-2: a pilot study
Source: Front Psychol. 2023 Nov 17;14:1244566. doi: 10.3389/fpsyg.2023.1244566 (PMC10690814; doi:10.3389/fpsyg.2023.1244566)
Supplement: Supplementary file 1 [file Data_Sheet_1.PDF]

Avaliação Canadiense da Literacia Física

(Versão Portuguesa).

O que pensas da atividade física?

Quando falamos sobre atividade física, referimos a quando estás em movimento, a brincar ou a exercitar-te. A atividade física é qualquer atividade que faz com que o teu coração bata mais depressa ou te faça ficar cansado durante algum tempo.

Por que estamos a fazer estas perguntas?

Queremos saber o que crianças, como tu, pensam sobre a atividade física, desporto e exercício físico.

Por favor lembra-te:

- Não há respostas certas ou erradas! Nós só queremos saber o que tu pensas.
- Se não souberes uma resposta, por favor escreve o que achares melhor.
- Não há limite de tempo, então, por favor, usa todo o tempo que precisares.

O que é mais parecido comigo?

Para cada pergunta, deves ler duas frases e fazer um círculo na frase que tu achas que é **MAIS PARECIDO CONTIGO.**

Experimenta no seguinte **EXEMPLO:**

**Algumas crianças têm um nariz na cara**	MAS	**Outras têm três narizes na cara**

Isso não deve ser muito difícil para tu decidires!
Uma vez que tu fizeste um circulo à volta da frase que mais se parece contigo, agora tens que decidir se para ti é **CONCORDO TOTALMENTE** ou **CONCORDO MAIS OU MENOS**

Aqui está outro exemplo para experimentar. Lembre-se, para responder à pergunta, precisa de duas coisas:

1. **Primeiro, um círculo na frase que mais se parece contigo.**

2. **Em seguida, marcar a caixa correta se, para ti, for CONCORDO TOTALMENTE ou CONCORDO MAIS OU MENOS**

NÃO EXISTEM RESPOSTAS CERTAS OU ERRADAS, APENAS DEVES DIZER O QUE ACHAS MAIS COMO PARECIDO CONTIGO!

Exemplo de pergunta nº 2:

○ **Algumas crianças gostam de brincar com computadores**<br>□ CONCORDO TOTALMENTE □ CONCORDO MAIS OU MENOS	**MAS**	○ **outras crianças não gostam de brincar com computadores**<br>□ CONCORDO TOTALMENTE □ CONCORDO MAIS OU MENOS

Agora estás pronto para começar a preencher este formulário. Lembra-te, em cada caixa precisas fazer um circulo no que é mais parecido contigo e, de seguida, marcar uma caixa para "totalmente" ou "mais ou menos" concordo. Demora o tempo necessário e faz todo o questionário com cuidado. Se tiveres alguma dúvida, é só perguntar! Se achas que estás pronto, podes começar agora.

CERTIFICA-TE DE PREENCHER CADA PÁGINA!

O que é mais parecido comigo?

○ **Algumas crianças não gostam de jogar jogos ativos ou jogos com movimento**<br>□ CONCORDO TOTALMENTE □ CONCORDO MAIS OU MENOS	**MAS**	○ **Outras crianças realmente gostam de jogar jogos ativos ou jogos com movimento**<br>□ CONCORDO TOTALMENTE □ CONCORDO MAIS OU MENOS

○ **Algumas crianças são boas em jogos ativos ou jogos com movimento**<br>□ CONCORDO TOTALMENTE □ CONCORDO MAIS OU MENOS	**MAS**	○ **Outras crianças acham que os jogos ativos ou jogos com movimento são difíceis de jogar**<br>□ CONCORDO TOTALMENTE □ CONCORDO MAIS OU MENOS

○ **Algumas crianças não se divertem muito praticando desporto.**<br>□ CONCORDO TOTALMENTE □ CONCORDO MAIS OU MENOS	**MAS**	○ **Outras crianças divertem-se a praticar desporto**<br>□ CONCORDO TOTALMENTE □ CONCORDO MAIS OU MENOS

○ **Algumas crianças são boas na maioria dos desportos**<br>□ CONCORDO TOTALMENTE □ CONCORDO MAIS OU MENOS	**MAS**	○ **Outras crianças sentem que não são boas no desporto**<br>□ CONCORDO TOTALMENTE □ CONCORDO MAIS OU MENOS

○ **Algumas crianças não gostam de praticar desportos**<br>□ CONCORDO TOTALMENTE □ CONCORDO MAIS OU MENOS	**MAS**	○ **Outras crianças gostam realmente de praticar desporto**<br>□ CONCORDO TOTALMENTE □ CONCORDO MAIS OU MENOS

○ **Algumas crianças aprendem a jogar jogos ativos ou jogos com movimento com facilidade**<br>□ CONCORDO TOTALMENTE □ CONCORDO MAIS OU MENOS	**MAS**	○ **Outras crianças acham difícil aprender a jogar jogos ativos ou jogos com movimento**<br>□ CONCORDO TOTALMENTE □ CONCORDO MAIS OU MENOS

Obrigado por nos dizer que crianças são mais parecidas contigo.

Temos apenas mais algumas questões. Por favor, vira a página.

Por que és ativo?

Rapazes e raparigas podem **ser ativos** ao fazer diversas coisas:

- Exercício (caminhadas, manter a forma ou aulas no ginásio)
- Brincar ao ar livre ou fazer coisas ativas (como brincar no parque)
- Desportos (como futebol, ténis, hóquei, dança ou natação)

Abaixo estão alguns motivos pelos quais tu podes ser ativo.

Por favor, lê cada frase e diz-nos o quão verdadeira é para ti.

Como te sentes ao ser ativo?

	Discordo muito	Discordo	Concordo	Concordo bastante	Concordo totalmente
Ser ativo é divertido	☐	☐	☐	☐	☐
Eu divirto-me a ser ativo	☐	☐	☐	☐	☐
Gosto de estar ativo	☐	☐	☐	☐	☐

Como te sentes por estar ativo?

A secção seguinte tem algumas frases que descrevem como as raparigas e os rapazes se sentem sobre SER ATIVO e FAZER COISAS ATIVAS (como jogos ativos ou jogos com movimento, jogar na rua e fazer desporto).

Por favor, lê cada frase e diz-nos o quanto se relaciona contigo.

	Nunca	Poucas vezes	Por vezes	Muitas vezes	Sempre
Quando se trata de jogar jogos ativos, acho que sou muito bom	☐	☐	☐	☐	☐
Acho que sou bom em atividades em comparação com outras crianças	☐	☐	☐	☐	☐
Quando se trata de ser ativo, tenho boas habilidades	☐	☐	☐	☐	☐

1. Quantos minutos por dia tu e as outras crianças devem fazer atividades físicas que façam com que o coração bata mais depressa e te faça respirar mais rápido, como andar rápido ou correr? Conta o tempo de atividade na escola e também quando estás em casa ou com os teus vizinhos.

a) 20 minutos.
b) 30 minutos.
c) 60 minutos ou 1 hora.
d) 120 minutos ou 2 horas.

2. Existem muitos tipos diferentes de aptidão física. Um tipo é chamado de resistência, ou aptidão aeróbica ou aptidão cardiorrespiratória. Aptidão cardiorrespiratória significa:

a) Quão bem os músculos podem empurrar, puxar ou esticar.
b) Quão bem o coração pode bombear sangue e os pulmões podem fornecer oxigênio.
c) Ter um peso saudável para nossa altura.
d) A nossa capacidade de praticar desportos que gostamos.

3. Força muscular ou resistência muscular significa:

a) Quão bem os músculos podem empurrar, puxar ou esticar.
b) Quão bem o coração pode bombear sangue e os pulmões podem fornecer oxigênio.
c) Ter um peso saudável para nossa altura.
d) A nossa capacidade de praticar desportos que gostamos.

4. Se quisesses SER MELHOR NUMA HABILIDADE DESPORTIVA (como chutar e apanhar uma bola), qual seria a melhor coisa a fazer?

a) Ler um livro sobre chutar e apanhar bolas
b) Esperar até ficar mais velho.
c) Tentar exercitar ou ser mais ativo.
d) Assistir a um vídeo, fazer uma aula ou pedir a um treinador que te ensine a chutar e apanhar a bola.

5. Faltam algumas palavras nesta história sobre a Maria. Escolhe as palavras na caixa para preencher as palavras que faltam na história. Cada palavra só pode ser usada para preencher um espaço em branco na história. Há mais palavras do que espaços em branco, portanto, nem todas as palavras serão usadas.

	**Divertida**	**Alonga**	**Resistência**
**Pulsação**	**Respiração**	**Flexibilidade**	**Boa**
	**Força**	**Mau**	**Desporto**

A Maria tenta ser ativa todos os dias. Correr todos os dias é bom para o coração e para os pulmões. A Maria acha que atividade física é ______ e também é ______. No treino da sua equipa desportiva ela faz mais corridas para melhorar a sua ______ . A equipa também faz exercícios como flexões e abdominais que aumentam a sua______. Ao arrefecer, ela ______ para melhorar sua flexibilidade e diminuir sua frequência cardíaca. Após o exercício, verifica a sua frequência cardíaca, que também é chamada de ______.

6. Durante a última semana (7 dias), quantos dias foste fisicamente ativo mum total de pelo menos 60 minutos por dia? Conta todo o tempo que gastaste a fazer atividades que aumentaram o teu ritmo cardíaco ou te fizeram respirar com dificuldade.

Tenho estado ativo durante **1** **2** **3** **4** **5** **6** **7** dias.

Nome: ______________________________________

Fale-nos de si...

Por favor, faça um círculo à volta de um número ou de uma palavra para cada pergunta.

Você é:

□ Menino

□ Menina

Em que mês é o seu aniversário?

Janeiro Fevereiro Março Abril Maio Junho Julho Agosto Setembro Outubro Novembro Dezembro

Qual é a sua idade?

5 6 7 8 9 10 11 12 13 14 15

Obrigado por responder às nossas perguntas!
